# Therapeutical and Pharmaceutical Notes

**Published:** 1900-03

**Authors:** W. J. Martin

**Affiliations:** Kankakee, Ill.


### THERAPEUTICAL AND PHARMACEUTICAL NOTES.

UNDER THE DIRECTION OF W. J. MARTIN, M.D.C.,
KANKAKEE, ILL.

REVISION OF THE UNITED STATES PHARMACOPŒIA. There has been called to meet in Washington, D. C., May 3, 1900, a convention to revise the *Pharmacopœia* of the United States of America. The members of this convention will be composed of delegates from the various medical and pharmaceutical associations of the United States which shall have been in existence for a period of five years previous to the date of the convention. The United States Army and Marine Hospital Service will also have representation in the convention.

While the *United States Pharmacopœia* in its present form appeals more directly to the pharmacist and practitioner of human medicine, it is nevertheless of some value as a guide to the preparation of the various medicinal compounds and new remedies used by the veterinarian.

There is at the present time much discussion going on among the members of the medical and pharmaceutical professions as to what drugs shall be admitted into the revised work. It is claimed by many that in order to make the work more popular with the members of the medical profession the *Pharmacopœia* should contain the maximum and minimum doses of the medicinal preparations therein contained. Why the *Pharmacopœia* should not contain such doses always appeared an enigma to the writer.

The introduction of new remedies or patented synthetics into the *Pharmacopœia* is a subject that has given rise to much discussion both for and against. However, the general opinion among pharmacists and medical men seems to be that the Committee on Revision will recommend the incorporation of a few of the more important synthetic drugs, such as are definite chemical compounds and whose extensive use in medicine and pharmacy justify such a course.

In its present composition the *United States Pharmacopœia* can scarcely be considered an indispensable work of reference to the American veterinarian, as in its compilation his profession is entirely ignored. This fact becomes very evident when the *United States Pharmacopœia* is compared with some of the European pharmacopœias, more especially the French. This pharmacopœia or

"Codex," as it is usually called, is considered the most complete work of its kind in existence. It contains in the form of an appendix a complete pharmacopœia of veterinary medicines, giving to our profession in that country the recognition it so richly deserves.

The time has come when the veterinary profession of North America should cease to depend upon foreign veterinary pharmacopœias, many of these being wholly unsuited to the requirements of the profession in this country. Again, many of these works have through lack of revision become obsolete, and thus in a certain sense are misleading to the students of veterinary pharmacy who consult them. In an endeavor to improve the status of American veterinary pharmacy the Executive Committee of the American Veterinary Association at its last meeting recommended that the president of the Association appoint a committee of three members who should endeavor to secure the incorporation in the next revised edition of the *United States Pharmacopœia*—in the form of an appendix—a pharmacopœia of veterinary medicine.

HAS THE BLADDER ANY POWER OF ABSORPTION? Barbiani (*La Riforma Medica*, August 11, 1899) has carried out a series of experiments on dogs and rabbits with relation to the above subject. On alternate days for an hour at a time he washed the bladder out with solutions of boric acid, phenol, sublimate, salicylic acid, antipyrine, quinine, cocaine, atropine, strychnine, and iodide of potassium (very slightly). In subsequent washings absorption took place, and the greater the number of washings the more marked the absorption. This absorption occurred without any alteration in the minute structure of the vesical epithelium (except in the case of the phenol and sublimate solutions, where slight changes in the superficial layer of the epithelium were noticed). The absorptive power was not increased by keeping a catheter permanently in the bladder.—*British Medical Journal*, October 21, 1899.

A FORMULA FOR THE HYPODERMATIC INJECTION OF ACOIN. Acoin, which is one of the new local anæsthetics made by synthesis, possesses the advantage that it produces anæsthesia rapidly, and that this anæsthesia is maintained from forty to fifty minutes. The following solutions may be used for hypodermatic purposes: ℞.—Acoin, 16 grains; chloride of sodium, 15 grains; distilled water, 3 ounces.—*Therapeutic Gazette.*

THE ROLE OF IRON IN FORMING BLOOD. A. Hoffmann, of Halle (*Munchener medicinische Wochenschrift*, 1899, No. 29), whose experiments have already proved the absorption and excretion of iron in the small intestine in man, and also in animals its excretion by the colon, thus confirming the results arrived at by Macallum, Hall, and others, in continuing his investigations as to the mode of action of the metal, has experimented on ninety-eight rabbits, on some made anæmic by blood-letting and others normally healthy, and in both cases with and without the administration of iron. Particular attention was paid to the bone-marrow, spleen, and lymphatic glands as specially concerned in blood-formation; but the liver, kidneys, and small and large intestine were also examined, the blood-corpuscles were counted, and the hæmoglobin estimated, etc.; and the various preparations of iron and hæmoglobin tested as regards qualitative and quantitative absorption. He found that all forms of iron were absorbed in the duodenum, and entered the circulation in transport cells combined with albuminous matter in a combination which had no toxic action. It could be so demonstrated in large quantities in the spleen, liver, and especially in the bone-marrow, where crowds of these iron-laden cells were present; in the tardy blood-stream, in the parenchyma itself, and in the network of vessels within it. This organ alone exhibited after blood-letting a corresponding regenerative activity—an active hyperplasia of its parenchyma. The restoration of the red-corpuscles was more rapid, the bone-marrow richer in its contents after the administration of iron, while the spleen and lymphatic glands showed no difference. Iron given without blood-letting caused some increase of the red cells circulating, and of the fat, but not of cell formation in the bone-marrow. The restoration of the hæmoglobin was not so complete; there was no apparent increase in the coloring-matter of the blood from the use of iron. In fact, iron has a stimulating action on the physiological activity of the bone-marrow, and accelerates the ripening and entrance into the circulation of the young cells therein produced as a nuclear erythrocytes. Special preparations of iron are unnecessary, of hæmoglobin irrational; the action of the iron depends entirely on the quantity of the metal absorbed.—*British Medical Journal.*

Tinctures Made from Fluid Extracts. The slipshod method of preparing tinctures by reducing a certain amount of fluid extract with alcohol and water and calling the mixture a tincture should be discountenanced by all members of the medical profession. Such preparations are not tinctures in any sense of the word, being merely dilute fluid extracts. To bear out this assertion we note that the Australian Government has lately issued a decree prohibiting pharmacists from preparing tinctures, decoctions, and infusions from fluid or solid extracts, giving as a reason that preparations thus prepared are not the same as preparations prepared in the usual pharmacopœial manner. Mr. W. R. Naville, in a paper read before the Texas Pharmaceutical Association on this subject, says: "It is clearly unreasonable to claim that the same tinctures can be had by extract dilution as by drug exhaustion. The practice of using fluid extracts for making tinctures should be condemned as inimical to the interests of pharmacy and legitimate medicine."

UNITED STATES DISPENSATORY. The eighteenth edition of this excellent work has lately been issued. The first *Dispensatory* was brought out in 1833, and since that time this standard work has been of the greatest value in spreading phamaceutical and botanical knowledge throughout the Western Hemisphere. The present edition contains about two hundred new drugs or combination of drugs more than the preceding edition, and to make room for these new acquisitions without increasing unduly the size of the work much old matter has been eliminated. Most of the new additions to the present edition are drugs of the synthetic class, and many of these are manufactured under foreign copyrighted names. While the propriety of introducing these trade-marked drugs into a standard work of reference for the medical and pharmaceutical professions may be questioned, it will have to be admitted that many of the preparations have acquired a permanent place in the armamentarium of the physician and veterinarian. The convenience of having such preparations grouped in such a work as the *Dispensatory* will be obvious to all. The library of every progressive veterinarian should contain a copy of this work.

DEHORNING FLUIDS. The patented dehorning fluids sold in the drug-trade under fanciful names consist merely of a strong solution of caustic potash, to which is added a small amount of carbolic acid.

HARD SETTING FOR PLASTER OF PARIS. Plaster of Paris is said to set much more efficiently with sulphate of potassium than with the chloride of sodium. The potash salt may be used in any strength. The stronger the solution the quicker the setting.

INSECTICIDES. *Flea-powder* : ℞.—Napthalin, 4 ounces ; starch, 12 ounces ; reduce to a fine powder. This is an excellent powder for the removal of fleas from cats and dogs by rubbing into the skin of the animal and letting the powder remain for a day or two, when the same can be removed by combing or giving a bath to which some infusion of quassia or quassia chips have been added. This treatment is equally efficient for lice and ticks, with which dogs as well as cats are afflicted.

*Wash for fleas and lice on animals:* ℞.—Napthalin, 1 ounce ; wood spirit, 5 ounces ; green soap, 5 ounces ; water, 20 ounces. Mix. Rub well into the hair and wash the animal the next day. Rarely more than one repetition is called for.

*Dog-wash:* ℞.—Soft soap, 2 ounces ; creolin, 1 to 2 ounces ; methylated spirit, 10 ounces ; water, to 20 ounces. Mix. Dissolve the soap and creolin in the spirit, and add the water gradually.

*Cattle-dip for ticks:* Dr. Norgaard, of the Bureau of Animal Industry, finds the following dip useful, immersion lasting one minute : ℞.—Sulphur, 86 pounds ; extra dynamo oil, 1000 gallons. Mix.—MYERS BROS., *Druggist.*

DISINFECTION OF THE SKIN AND HANDS. Dr. Mikulicz (*Deut. med. Woch.*) recommends the German official soap spirit for this purpose, especially in country practice and in military service, where the precautionary measures available, *e. g.*, in a large hospital, are not practical. The spiritus saponatus is prepared with 6 parts of olive oil, 7 of liquor potassa, 30 of alcohol, and 17 of water. Spores of staphylococcus aureus introduced on balls into this spirit were killed in one-half minute, while in 0.1 per cent. solution of hydrogen dioxide they were not killed before ten minutes. If the balls were first moistened in water sterilization took two minutes. Similar results were obtained with other microörganisms. Experiments made on the hands, including observations after they have been infected by contact with septic wounds, gave absolute sterility in 40 per cent. Soap spirit causes no irritation, acts thoroughly, its effect lasts some time, it acts quickly, is harmless and odorless.

GOLINDRINERA, OR EUPHORBIA PROSTRATA, FOR SNAKE AND INSECT BITES. Dr. E. Hammond, of Noria, Mexico, writing in the *Medical Brief*, says about euphorbia prostrata, also called golindrinera (corrupted into " golandrina ") that its reputed virtues are well merited. "I have been using it for the last thirty years, and it has never disappointed me as an antidote to the poison of serpents and all kinds of venomous animals with only one exception, and that in a small spider, called here the 'uvar.' I use the whole plant and root. I make a saturated solution or tincture with dilute alcohol, and give from one-half to one ounce every thirty to sixty minutes, according to circumstances, and apply same to wound. This is for small animals such as scorpions, centipedes, etc. For rattlesnake bites I give as much as two ounces every hour or two, and help it by keeping the wound well covered with the tincture of iodine. I use the plant dried and powdered in making the tincture. With me it has never proved either emetic or cathartic." This plant is mentioned in the *United States Dispensatory*, where the euphorbia ocellata of the Pacific coast also is said to be used as an antidote for snake-bites.

NAFTALAN IN OBSTINATE SKIN DISEASE. This drug is described by Poyntz-Wright (*Therapist*), who has found it extremely useful in various chronic skin affections. It is procured from a crude naptha found in the Caucasus at a place called Naftalan, from which it acquires its name. It occurs in the form of a greenish-black mass, insoluble in water or glycerin, but mixing easily with fat, and soluble in ether and chloroform. It will never become rancid, and is non-poisonous. In chronic eczema it has given most satisfactory results, the inflammatory condition subsiding quickly, and the exudation drying up with equal rapidity, added to which there was almost immediate relief from the distressing burning and stinging sensations. It has also been used with success in psoriasis inveterata and pityriasis versicolor, and effectually prevents pealing off of the epidermis in tender skins that are affected by sea-water and the hot sun. It is also recommended in burns, erysipelas of the face, and as a local application in rheumatism and gout.—*Medical Standard.*

SODIUM BICARBONATE FOR SUPPURATING WOUNDS. Ange and Casteret (*La Presse Med.*) say that bicarbonate of sodium used in a 2 per cent. solution is an excellent treatment for suppurating wounds. They have used it in some sixty cases with great satisfaction. The solution is not bactericidal, but acts in a wound as an antiseptic, probably because its alkalinity preserves the tissues and puts them and the blood in the best condition to overcome the germs. Moreover, the healing of a wound, according to Preobrajenski, is not exclusively due to the destruction of germs. It depends upon the capillary circulation in the wound. If the liquids in the wounded area pass from within the body outward they carry off toxins, if they pass from without inward general infection follows. This view has been proven by a number of experiments. Bicarbonate of soda by dissolving the fatty matter of the skin stimulates the secretions through all the tissues in a vital condition. It is in a certain sense, therefore, a physiological dressing.

Camphorated Oil a Carbolic-acid Antidote. Alvarez (*Zeits. f. Phar.*) reports a case of successful recovery from carbolic-acid poisoning by the administration of camphorated oil.—*Western Druggist.*

Inoculation Against Plague. In an address before the Royal Society in London, Haffkine (*Lancet*) cites some results from the use of his prophylactic serum in the modified form now used. For instance, at the Byculla jail there were twelve cases of plague and six deaths among 172 uninoculated prisoners, and only two cases with no deaths among 147 inoculated prisoners. At Umerkadi jail there were ten cases and six deaths among 127 uninoculated, and only three cases with no deaths among 147 inoculated. Again, at the village of Undhera there were twenty-seven cases and twenty-six deaths among sixty-four uninoculated inhabitants, and eight cases with three deaths among seventy-one inoculated inhabitants. Other observations on a larger scale were quoted, such as those at Lanowlie, Kirkee, Damaon, Hubli, Dharwar, and Gadag, and in each the difference in the mortality from the plague between the inoculated and the uninoculated was estimated to average over 80 per cent. Further, it appears from data collected in the larger hospitals that the case-mortality among the inoculated admitted for plague is at least 50 per cent. lower than among the uninoculated. The accuracy of these results is vouched for by numerous independent observers. Even opponents have been compelled by the logic facts to admit that Mr. Haffkine's system has triumphed.—*Ibid.*

Discolored Eserine Solutions. Solutions of eserine easily become discolored. According to Hallauer (*Zeit. f. Augenhl.*) their mydriatic action is not diminished thereby, but secondary results are produced which render their actions undesirable. The change may be prevented by adding 4 per cent. of boric acid, which does not interfere with the physiological action of the alkaloid. The sulphate is said to be less stable than the salicylate.

